# Transforming global approaches to chronic disease prevention and management across the lifespan: integrating genomics, behavior change, and digital health solutions

**DOI:** 10.3389/fpubh.2023.1248254

**Published:** 2023-10-13

**Authors:** Shane A Thomas, Colette J Browning, Fadi J Charchar, Britt Klein, Marcia G. Ory, Henrietta Bowden-Jones, Samuel R. Chamberlain

**Affiliations:** ^1^Vice Chancellor’s Office, Federation University, Ballarat, VIC, Australia; ^2^Institute of Health and Wellbeing, Federation University, Ballarat, VIC, Australia; ^3^Health Innovation and Transformation Centre (HITC), Federation University, Ballarat, VIC, Australia; ^4^Center for Community Health and Aging, Texas A&M University, School of Public Health, College Station, TX, United States; ^5^National Problem Gambling Clinic, London, United Kingdom; ^6^Department of Psychiatry, University of Cambridge, Cambridge, United Kingdom; ^7^Faculty of Brain Sciences, University College London, London, United Kingdom; ^8^Department of Psychiatry, Faculty of Medicine, University of Southampton, Southampton, United Kingdom; ^9^Southern Gambling Service, and Southern Health NHS Foundation Trust, Southampton, United Kingdom

**Keywords:** chronic diseases, prevention, genomics, risk prediction, behavior change, digital health, healthy aging

## Abstract

Chronic illnesses are a major threat to global population health through the lifespan into older age. Despite world-wide public health goals, there has been a steady increase in chronic and non-communicable diseases (e.g., cancer, cardiovascular and metabolic disorders) and strong growth in mental health disorders. In 2010, 67% of deaths worldwide were due to chronic diseases and this increased to 74% in 2019, with accelerated growth in the COVID-19 era and its aftermath. Aging and wellbeing across the lifespan are positively impacted by the presence of effective prevention and management of chronic illness that can enhance population health. This paper provides a short overview of the journey to this current situation followed by discussion of how we may better address what the World Health Organization has termed the “tsunami of chronic diseases.” In this paper we advocate for the development, validation, and subsequent deployment of integrated: 1. Polygenic and multifactorial risk prediction tools to screen for those at future risk of chronic disease and those with undiagnosed chronic disease. 2. Advanced preventive, behavior change and chronic disease management to maximize population health and wellbeing. 3. Digital health systems to support greater efficiencies in population-scale health prevention and intervention programs. It is argued that each of these actions individually has an emerging evidence base. However, there has been limited research to date concerning the combined population-level health effects of their integration. We outline the conceptual framework within which we are planning and currently conducting studies to investigate the effects of their integration.

## Introduction

The world is slowly emerging from one of its most challenging periods in modern human history with the COVID-19 pandemic and its aftermath. Before the COVID pandemic the focus was on Chronic/Non-Communicable Diseases (NCDs). The WHO President (1) then warned about the growth in chronic disease burden. The significance of NCDs was recognized in the United Nations 2030 Agenda for Sustainable Development, which set targets to, *“reduce by one third premature mortality from noncommunicable diseases through prevention and treatment, and promote mental health and well-being”* (2). Unfortunately, it is now clear that COVID-19 has contributed to major upticks in underlying and consequential chronic illnesses and diseases from an already high base (3). We now seem to be in an even worse position than before. We have a syndemic driven by the existing chronic disease pandemic overlaid by the newer COVID-19 pandemic (4). A key challenge in the syndemic is to provide adequately resourced and well-trained public and clinical health workforces (5–7).

### The burden of chronic diseases and illness in the human population

The burden of chronic illness globally has increased over time, accounting for the major part of global disease burden (8, 9). The epidemiology of chronic disease burden varies from country to country but most countries whether developed or developing have high chronic disease burden. The Lancet global burden studies chronicle the burden of 369 diseases and injuries in 204 countries and territories. They show that chronic conditions caused 74% of all deaths worldwide in 2019, rising from 67% of deaths in 2010. The mortality data reflect high prevalence’s of chronic conditions across populations. For example, in Australia, the Australian Institute of Health and Welfare (10), has noted 47% of Australians have at least one chronic disease with 20% having 2 or more. 51% of hospitalizations involve chronic disease, 90% of deaths and disease burden is borne disproportionately by adults of lower SES and those living in remote areas. These results are consistent with those globally and in many countries. The USA NIH (11) has noted “*currently, some 50% of the US population has a chronic disease, creating an epidemic, and 86% of healthcare costs are attributable to chronic disease.”* In the United Kingdom a similar epidemiological pattern is evident with close links between traditional physical chronic diseases and associated mental health disorders (12). These studies show increased prevalence across the lifespan threatening healthy aging.

### The contribution of mental health disorders, including addictions, to population disease burden

Mental health disorders including addictions are now an increasing challenge facing humanity (13, 14) with 20 percent of global disease burden. One might be forgiven for labeling recent decades as the “Age of Addiction” (15) with the addiction “traditionals” of alcohol, cannabis, and other substances and new synthesized agents overlaid by newly recognized behavioral addictions (16) (see the WHO ICD (17) and AMA DSM) (18). Of the behavioral addictions, the first was Gambling Disorder (GD) – which continues to be neglected in terms of research and development of new interventions. GD is linked to high levels of mental and physical health comorbidities, health economic costs, homelessness, and suicidality (19, 20). In addition to addictions, there is strong growth in the population prevalence’s of mood disorders and anxiety (21). Interpreting the true growth in mental health disorders has some nuances. Recognition of the importance of mental health disorders (12, 22) has also led to increased prominence in national health surveys and public health epidemiological studies. These changes in population health study content make it difficult to assess the true extent of the underlying growth in the population prevalence of such disorders. Another problem is that the most pressing mental health conditions are seldom appropriately measured in public health epidemiological studies – especially GD. These issues are examples of “what gets measured gets managed” and contrariwise. There are many instances where inclusion of measures has improved health services and policy making (23). For example, mental health measures are now an increasingly prominent component of clinical datasets and such conditions are receiving greater clinical effort and funding. The same is true for chronic diseases in general which are now centrally located in health policy and service design in many countries.

The evidence base provided by disease burden and epidemiological studies strongly reinforces the WHO’s alarm. What therefore is to be done? How can the global chronic diseases/long-term conditions pandemic be better addressed? Below we recommend an integrated population-level approach involving (1) large-scale measurement of polygenic and multifactorial risk factors in order to develop and rigorously validate clinically useful prediction tools and algorithms; (2) early and sustained, effective management of chronic diseases using advanced behavior change interventions and (3) digital health approaches to improve the efficiency and reach of interventions and health services at a wider population level.

### Development, validation, and deployment of risk prediction tools using polygenic and multifactorial risk data

Early identification of people at risk of chronic illness and early intervention are key to reducing population chronic disease burden. Unfortunately, this obvious game changer is infrequently implemented in many public health regimens. We now have much better technology available to develop and validate evidence-based risk prediction tools and algorithms, and to demonstrate their value by leveraging digital tools that can be embedded within at-scale screening and treatment programs. We already have the technology to develop and validate useful risk prediction tools and algorithms but we contend that we are not yet systematically conducting such research and implementing resulting tools at the required pace in large-scale studies as outlined in the recent International Common Disease Alliance Polygenic Risk Score Task Force report (24). The first contact with many people with chronic disease risk is after they have already developed it (25, 26). For mental health disorders, delays in presentation and intervention can be particularly long, adding to the burden of disease. Obsessive-compulsive disorder (OCD) is one of the top ten leading causes of disability in the developed world and has a typical duration of untreated illness of 10 years (27). While less well studied, a similar duration of untreated illness has been reported for gambling disorder – around 9 years in affected individuals presenting for treatment (28).

Polygenic and multifactorial risk prediction can play a major role in delivering early warning of impending chronic diseases (29) including traditional chronic diseases such as cardiovascular disorders (30), metabolic disorders (31), and cancers (32). While more research is certainly needed, initial data suggest some promise for mental health disorders (33) including gambling disorder and newer concepts such as gaming disorder (34, 35). Wider implementation of such strategies has the potential to drive down the costs of what are now mature and proven technologies, but already they are affordable. The costs associated with inaction with chronic diseases are substantial (36). The potential cost reductions in health care costs and the net benefits of prevention, early detection and intervention are well established in principle. It is our view that humanity cannot afford to further delay polygenic and multifactorial risk prediction, early diagnosis and intervention.

Beyond the need for data collection and linked rigorous validation, to evaluate the potential value of incorporating polygenic and multifactorial risk prediction into routine practice, the global public health and health care workforces need to be trained to use these tools, to effectively communicate the meaning of risk and risk management to the community. Such training is relatively common for some conditions but neglected in other conditions. In the United Kingdom, there has been recent work to promote the wider use of genomics in General Practice (37, 38).

### The prevention and management of chronic diseases

Lifestyle risk factors make a major contribution to the chronic disease burden over the life course. Many of the same risk factors contribute to multiple chronic diseases. Given the high contribution of these behavioral risk factors to multiple chronic diseases, and, that these diseases comprise such a high proportion of total disease burden, it is obvious that public health and clinical workforces need strong chronic disease program prevention skills. Inclusion of such matters in the medical and clinical health curriculums is an essential and welcome innovation to contemporary chronic disease management (39–41).

It is important to understand that treatment for the various chronic diseases must follow recognized evidence based clinical and population health prevention guidelines. Study of these guidelines show that there is a high degree of commonality in the risk factors that contribute to chronic diseases. Advanced behavior changes skills facilitate more effective prevention and management of chronic disease.

Some of this work on expanding training across health systems has been conducted by members of the authorship team. The training has been directed at public health practitioners and clinical workers in public health programs. The Happy Life Club originated in Australia and was then translated to China in various major cities and provinces where it has grown strongly (42–44). Initial economic evaluation (45) showed that incremental benefit for each patient corresponded to $AUD 16,000 over an 18-month period. The 2020 frontiers special issue devoted to chronic disease and aging (46, 47) built on other work concerning Chronic Disease Management (CDM) programs (48). The Club program trains public health practitioners and clinicians to prevent and manage chronic conditions using Motivational Interviewing (MI) principles (49).

The Happy Life Club coach training program has been (50–52) studied as the subject of evaluations and the program was the subject of a large Randomized Controlled Trial (53) and is a World Bank recommended intervention (54). While MI is a central part of the program, rigorous outcome measurement using validated tools and patient-centered care principles are also key components. These techniques are more broadly applicable across a range of chronic diseases including mental health and addiction disorders (55–57) in controlled trials. It would seem sensible to conduct clinician and public health training so that MI techniques can be more widely applied in public health settings with the aim of further reducing the impact and burden of chronic mental health symptoms.

### Digital health platforms: improving population reach and program efficiency

Digital health platforms hold the potential to facilitate Chronic Disease Management across the lifespan at scale in health systems. A large-scale review of digital health platforms was conducted by WHO and the Cochrane Collaboration to develop the Digital Interventions for Health System Strengthening guideline (58). Eleven new Cochrane reviews were included in the guideline. The guideline highlighted different applications of digital health including conventional public health programs, prevention, clinical delivery and back-office programs. It also highlighted the need for the collection of more evidence for these platforms. A 2022 European review (59) investigating the cost-effectiveness of digital health interventions concluded that the evidence was not yet sufficient to return a positive or negative conclusion. We are committed to providing effective digital health support to public health and clinical workers for chronic disease prevention and intervention programs. The goal is to address the knowledge as to how such tools may be best applied (60–63).

Of course, as noted, it is important to be mindful that specific tools are needed for specific purposes and generalizing across all tools is of limited value. Examples of recent digital tool development work in the field of alcohol use include a tool that can estimate weekly alcohol intake based on responses to the extended AUDIT questionnaire; and a web-app brief intervention to raise awareness about the impact of alcohol on breast cancer in a breast cancer clinic setting (potentially modifiable risk factors account for around 25% of breast cancer cases (64, 65)). Another example is that in work led by UK members of our group, we have developed and are piloting a digital tool for NHS gambling treatment services, which collects validated assessment and outcome data from affected individuals and generates readily interpretable summary reports, which are then discussed by the clinician and their patient. This approach could have potential advantages in terms of streamlining the clinical assessment process, fostering early and sustained patient engagement, and improving quality and volume of research data to improve care pathways.

Overall, digital tools are at different levels of development and only some could be deemed sufficiently validated for current widespread use. However, we feel these examples highlight the potential utility of such tools for public health prevention and interventions, in the management and mitigation of chronic diseases. Smart technologies are now available with much-improved access. Recent data shows that access to the Internet of Things will grow from 15 billion to 30 billion devices in the next 7 years (66). Eight billion of these devices will be smartphone connections (67). It is well understood that the availability of smart devices has been less among economically disadvantaged groups. However, this is not cause to deny the obvious advantages of using smart device technologies in global prevention and management of chronic disease of the majority of people.

### The importance of rigorous evaluation and validation of approaches to chronic disease management and prevention

As previously stated, there is significant evidence for the efficacy of polygenic and multifactorial risk screening, strong evidence for the efficacy of advanced behavior change principles and a developing evidence base for the efficacy of digital health platforms for some conditions and populations (68–70). Rigorous evaluation of the impact of these approaches in combination and their efficacy across varied populations and chronic diseases and disorders is required. The systematic reviews that have been done of the cost-effectiveness of programs to address chronic diseases show significant advantages (71) but many studies do not include adequate economic analysis or active comparator/control conditions.

The conduct of health economics modeling is a key plank of the valuation of chronic disease management programs. Using tools such as the EuroQoL suite and the use of look up tables developed from major studies credible Disability Adjusted Life Years (DALY) and Quality Adjusted Life Years (QALY) estimations can be constructed from the data (72).

There is the technical issue of what methods of economic evaluation ought to be used to assess the costs and benefits of the study outcomes based on the collected data (73). Many studies use an ICER (incremental cost-effectiveness ratio) or an INMB (incremental net monetary benefit) of the intervention compared with usual programs. Both ICER and INMB methods have limitations, but ICER is currently more widely used. INMB has some appealing aspects. A key one is that it is couched in terms of dollar values whereby costs and benefits are expressed in the same dollar value units. This provides not only ease of interpretation but also the ability to use it to enable direct comparison across different programs. All studies in the program will conform to the design principles outlined in the Bias in Economic Evaluation (ECOBIAS) standards (74) and Consolidated Health Economic Evaluating Reporting Standards (CHEERS) (75) to guide the reporting of study outcomes and rigorous design.

### Summary of the suggested multidisciplinary population chronic disease prevention and management approach INTEGRATE

**Figure 1:**
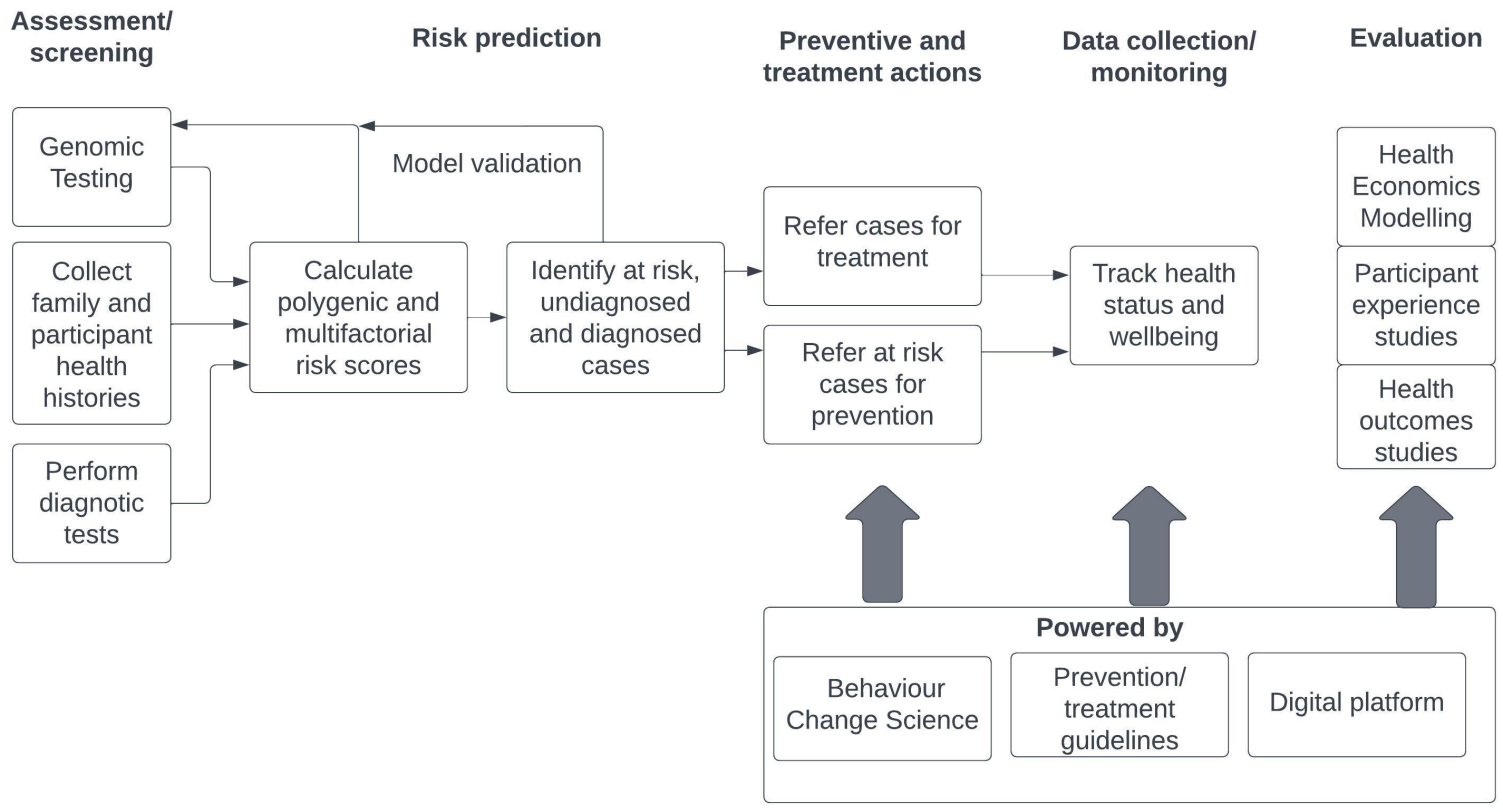
INTEGRATE program logic map.

The following chart outlines the logic of the INTEGRATE program approach that aims to enhance improved health outcomes across the lifespan through, prevention, earlier detection and more effective intervention. It is intended that the INTEGRATE model will be applied to a range of chronic physical and mental health conditions at a population level, reflecting the high degree of multi-morbidity identified in population and clinical studies. The INTEGRATE model does not replace guidelines-driven programs and interventions. It provides an organizing framework for important strategic disease risk prediction data based on genomics science and multi-factorial risk assessment, supports public health workers and clinicians using their disciplinary evidence-based prevention/ treatment guidelines to deal with a range of chronic illnesses by enhancing their behavior change skills and assists with the cost-effective delivery of treatment by utilizing advanced digital health platform capabilities. The public health and clinical workers are augmented by powerful support tools (Figure 1).

The INTEGRATE model seeks to combine polygenic genomic and diagnostic testing and history data for target chronic illnesses to identify sub-populations that are low risk, at risk and with diagnosed and undiagnosed conditions. Those with high risk but no diagnosed condition are referred for preventive actions to lower their risk. Those with diagnosed conditions are referred into treatment programs to improve health and wellbeing. All cases in the preventive and treatment programs may be tracked to assess their ongoing health status and wellbeing. These program actions are powered by behavior change science and prevention and treatment guidelines pertinent to their chronic illnesses assisted by digital platform technologies. It is intended that cases and at-risk community members will be detected and enter earlier preventive and treatment programs. Health economics, participant experience and health outcomes studies will be used to evaluate program effectiveness and efficacy. It is intended that health care costs will be lowered, risk reduced, and outcomes improved by the application of this model through its integration into public health and clinical programs targeting chronic diseases.

We have now set ourselves the task of evaluating the efficiency and effectiveness of the INTEGRATE approach we have described for integrated prevention and effective interventions across a range of chronic diseases. We believe that this approach addresses several key problems. Although there have been many exhortations of the virtues of prevention and early invention among at-risk populations, the genomic technologies that practically and expediently this approach are recent, but in some cases are now sufficiently developed to trial and use at scale. However, we must take an evidence-based and skeptical approach using health economics and rigorous clinical efficacy evidence including appropriate control conditions (where feasible). Behavior change science has been with us for several decades, but its power has not been fully implemented due to lack of contemporary training throughout the public health and clinical workforces. This science does not replace, for example, pharmacological and other interventions, it complements and augments them.

We feel these promising technologies are within our grasp and now we have the duty of evaluating and implementing them in an effective integrated way to advantage the targeted populations within our communities. This integrated approach has considerable promise for promoting population health, healthy aging and reducing the current burdens of health care. We also have a commitment to not artificially separate “physical” and “mental” health conditions in a context where they are so demonstrably interdependent.

## Data availability statement

The original contributions presented in the study are included in the article/supplementary material, further inquiries can be directed to the corresponding author.

## Author contributions

ST and CB conceptualized the paper. ST drafted the paper and had final editorial approval. CB assisted with the drafting of the paper and provided editorial input. FC contributed to the genomic sections of the paper. BK contributed to the digital health sections of the paper. MO provided a public health perspective to the drafting of the paper. HB-J and SC assisted with drafting of the mental health sections of the paper. All authors provided editorial comments in addition to their substantive contributions.
